# Sparse generalized linear model with *L*_0_ approximation for feature selection and prediction with big omics data

**DOI:** 10.1186/s13040-017-0159-z

**Published:** 2017-12-19

**Authors:** Zhenqiu Liu, Fengzhu Sun, Dermot P. McGovern

**Affiliations:** 1 Samuel Oschin Comprehensive Cancer Institute, Cedars-Sinai Medical Center, Los Angeles, 90048 CA USA; 2 Molecular and Computational Biology Program, Department of Biological Sciences, University of Southern California, Los Angeles, 90089 CA USA; 3 Foundation Inflammatory Bowel & Immunobiology Research Institute, Cedars-Sinai Medical Center, Los Angeles, 90048 CA USA

**Keywords:** Sparse modeling, *L*_0_ penalty, Big data mining, Multi-omics data, GLM, Classification, Suboptimal debulking

## Abstract

**Background:**

Feature selection and prediction are the most important tasks for big data mining. The common strategies for feature selection in big data mining are *L*_1_, SCAD and MC+. However, none of the existing algorithms optimizes *L*_0_, which penalizes the number of nonzero features directly.

**Results:**

In this paper, we develop a novel sparse generalized linear model (GLM) with *L*_0_ approximation for feature selection and prediction with big omics data. The proposed approach approximate the *L*_0_ optimization directly. Even though the original *L*_0_ problem is non-convex, the problem is approximated by sequential convex optimizations with the proposed algorithm. The proposed method is easy to implement with only several lines of code. Novel adaptive ridge algorithms (*L*_0_ADRIDGE) for *L*_0_ penalized GLM with ultra high dimensional big data are developed. The proposed approach outperforms the other cutting edge regularization methods including SCAD and MC+ in simulations. When it is applied to integrated analysis of mRNA, microRNA, and methylation data from TCGA ovarian cancer, multilevel gene signatures associated with suboptimal debulking are identified simultaneously. The biological significance and potential clinical importance of those genes are further explored.

**Conclusions:**

The developed Software *L*_0_ADRIDGE in MATLAB is available at https://github.com/liuzqx/L0adridge.

**Electronic supplementary material:**

The online version of this article (doi:10.1186/s13040-017-0159-z) contains supplementary material, which is available to authorized users.

## Background

Integrating multilevel molecular and clinical data to design preventive, diagnostic, and therapeutic solutions that are individually tailored to each patient’s requirements is the ultimate goal of precision medicine. However, the huge number of features makes it neither practical nor feasible to predict clinical outcomes with all omics features directly. Thus, selecting a small subset of informative features (biomarkers) to conduct association studies and clinical predictions has become an important step toward effective big data mining. Statistical tests or univariate correlation analysis for feature selection ignore the interacting relationship among genes. To evaluate the predictive power of the features, one appealing approach for feature selection is *L*_0_ regularized sparse modeling, which penalizes the number of nonzero features directly. *L*_0_ is known as the most essential sparsity measure and has nice theoretical properties. However, it is computational impossible to perform an exhaustive search when analyzing omics data sets with millions of features. *L*_0_ penalized optimization is known to be NP-hard in general (Lin et al. 2010).

One common strategy for feature selection is to replace the non-convex *L*_0_ with the *L*_1_ norm. *L*_1_ is a convex relaxation and loose approximation of *L*_0_. Although *L*_1_ penalized sparse models [1] can be solved efficiently, the estimators with *L*_1_ are penalized too much and asymptotically biased. In addition, *L*_1_ inclines to select more spurious features than necessary, and may not always choose the true model consistently [2]. Theoretically, *L*_1_ never outperforms *L*_0_ by a constant [3]. Depending on the location of true optimum, *L*_1_ may perform much worse than *L*_0_ [4, 5]. As a result, the convex relaxation techniques have been shown to be suboptimal in many cases [6]. More recent approaches aimed to reduce bias and overcome discontinuity include the non-convex SCAD [7] and MC+ [8]. However, none of the existing algorithms directly approximate the *L*_0_ optimization problem. Either SCAD or MC+ has been rarely used for feature selection in big data analytics because of their computational intensity with multiple tuning parameters. On the other hand, recent research works including ours show that sparse regression models with *L*_0_ penalty (local solution) outperforms *L*_1_ (global solution) by a substantial margin [5, 9–11].

Debulking cytoreductive surgery is a standard treatment for ovarian cancer. The goal of debulking is to remove as much visible cancer as possible. However, if tumor nodules have invaded vital organs, surgeons may not be able to remove them without compromising the patient’s life. Leaving tumor nodules larger than 1 cm is defined as suboptimal debulking (cytoreduction). It has been shown that suboptimal debulking is associated with reduced chemosensitivity and poor survival in ovarian cancer. Biomarkers derived from multi-omics data may help physicians decide which patients should undergo surgery and which should be treated with chemotherapy first [12–14]. Identifying biomarkers from multi-omics data has been an exciting but challenging task. Sparse modeling is one of the important approaches for simultaneous phenotype prediction and biomarker identification. In this paper, we propose a *L*_0_ penalized generalized linear regression (GLM) for feature selection and prediction. Adaptive ridge algorithm (*L*_0_ADRIDGE) is developed to approximate *L*_0_ penalized GLM with sequential convex optimization and is efficient in handling ultra high-dimensional omics data. The proposed method outperforms other cutting-edge convex and non-convex penalties including *L*_1_, SCAD and MC+ with simulations. When applied to the important suboptimal debulking prediction problem in ovarian cancer, the proposed approach identifies multilevel molecular signatures through mining methylation, microRNA and mRNA expression data jointly from TCGA. The identified molecular signatures are further evaluated using public databases.

## Materials and methods

Given an input *X*_*N*×*P*_, where *N*≪*P*, and output *Y*, we have a generalized linear model with canonical link in the following form:

$$ E(Y|X)=\mu = G({\theta}),\;\;\; \text{and} \;\;\; {\theta} =X {\beta}, $$

where *G* is a canonical link function. Different link functions lead to different models. For instance, a logit link function leads to logistic regression, while an exponential link function leads to Poisson regression.

### *L*_0_ penalized GLM

The distribution of Y in GLM is assumed to be from the exponential families with the following probability (density) function:

$$f(Y, {\theta}, \phi) = \exp\left\{\frac{Y{\theta}-B({\theta})}{A(\phi)} + C(Y, \phi)\right \}, $$

where *ϕ* is a dispersion parameter, and different functions *A*(∗), *B*(∗) and *C*(∗) are for different distributions *Y* [15]. The corresponding mean and variance are:

$$ E(Y) = \mu = B'({\theta}), \textrm{and }\; Var(Y) = V(\mu)A(\phi) = B^{\prime\prime}({\theta})A(\phi), $$

where *V*(*μ*)=*B* ^′′^(*θ*). Let *Y*=[*Y*_1_,…,*Y*_*N*_]^*t*^, *X*=[**x**_1_,**x**_2_,…,**x**_*N*_]^*t*^, and *μ*=[*μ*_1_,…,*μ*_*N*_]^*t*^, so $\mu _{i} = G({\theta }_{i}) = G\left (\mathbf {x}_{i}^{t} {\beta }\right)$ and ${\theta }_{i} = \mathbf {x}_{i}^{t} {\beta } $. The log-likelihood of Y is

$$\begin{array}{*{20}l} L(Y, \mu, \phi) & = \sum\limits_{i=1}^{N} \log f(Y_{i},{\theta}_{i}, \phi) \\ & = \sum\limits_{i=1}^{N}\left\{\frac{Y_{i}{\theta}_{i} -B({\theta}_{i})}{A(\phi)}-C(Y_{i}, \phi) \right\}. \end{array} $$

Dropping the constants *A*(*ϕ*), and *C*(*Y*_*i*_,*ϕ*), we have the simplified log likelihood as follows:

$$ L(Y, \mu) = \sum\limits_{i=1}^{N}\{Y_{i}{\theta}_{i} - B({\theta}_{i})\}. $$

Hence, *L*_0_ penalized error function to minimize is

1 $$\begin{array}{*{20}l} \underset{ {\beta} }{{\arg\min}} E & = \underset{ {\beta} }{{\arg\min}}\left\{ -L (Y, \mu) + \frac{ {\lambda} }{2} | {\beta} |_{0}\right\}  \\ & = \underset{ {\beta} }{{\arg\min}} \left\{\sum_{i=1}^{N} [B({\theta}_{i}) -Y_{i}{\theta}_{i}] +\frac{ {\lambda} }{2} | {\beta} |_{0} \right\}, \end{array} $$

where $| {\beta } |_{0} = \sum _{j=1}^{P} I({\beta }_{j} \neq 0)$ is the number of nonzero elements in *β*, *μ*_*i*_=*G*(*θ*_*i*_) and ${\theta }_{i} = \mathbf {x}_{i}^{t} {\beta } $. If we define $\frac {0}{0} = 0$, then $| {\beta } |_{0} = \sum _{j} I({\beta }_{j} \neq 0) = \sum _{j} \frac { {\beta }_{j}^{2}}{ {\beta }_{j}^{2}}$. Equation (2) is equivalent to

2 $$\begin{array}{*{20}l} \underset{ {\beta} }{{\arg\min}} E & = \underset{ {\beta} }{{\arg\min}}\{ -L (Y, \mu) + {\lambda} | {\beta} |_{0}\}  \\ &= \underset{ {\beta} }{{\arg\min}} \left\{\sum\limits_{i=1}^{N} \left[B({\theta}_{i}) -Y_{i}{\theta}_{i}\right] +\frac{ {\lambda} }{2} \sum\limits_{j=1}^{P}\frac{ {\beta}_{j}^{2}}{ {\beta}_{j}^{2}} \right\}, \end{array} $$

which is equivalent to the following system:

3 $$\begin{array}{*{20}l} \underset{ {\beta} }{{\arg\min}} E &= \underset{ {\beta} }{{\arg\min}}\{ -L (Y, \mu) + {\lambda} | {\beta} |_{0}\}  \\ &= \underset{ {\beta} }{{\arg\min}} \left\{\sum\limits_{i=1}^{N} [B({\theta}_{i}) -Y_{i}{\theta}_{i}] +\frac{ {\lambda} }{2} \sum\limits_{j=1}^{P}\frac{ {\beta}_{j}^{2}}{\eta_{j}^{2}} \right\},  \\ \eta & = {\beta}. \end{array} $$

Given *η* and ${\theta }_{i} = \mathbf {x}_{i}^{t} {\beta } $, the derivative of *E* w.r.t. *β* is

$$\begin{array}{*{20}l} \nabla E &= \sum\limits_{i=1}^{N}\left[B'({\theta}_{i}) -Y_{i}\right]\frac{\partial {\theta}_{i}}{\partial {\beta}} + {\lambda} {\beta} \oslash \eta^{2} \\ & = \sum\limits_{i=1}^{N}\left[B'({\theta}_{i}) -Y_{i}\right]\mathbf{x}_{i} + {\lambda} {\beta} \oslash \eta^{2}, \end{array} $$

where ⊘ indicates element-wise division. The Hessian matrix is

$$ H({\beta}) = \sum\limits_{i=1}^{N} B^{\prime\prime}({\theta}_{i})\mathbf{x}^{t}\mathbf{x} + {\lambda} \oslash\eta^{2}. $$

Let

$$D =\left[ \begin{array}{cccc} \eta_{1}^{2} & 0 &\ldots & 0 \\ 0 & \eta_{2}^{2} & \ldots & 0 \\ \vdots & \vdots &\ddots & \vdots \\ 0 & 0 &\ldots & \eta_{P}^{2} \end{array}\right], \; \text{and} \; V =\left[ \begin{array}{cccc} V_{1} & 0 &\ldots & 0 \\ 0 & V_{2} & \ldots & 0 \\ \vdots & \vdots &\ddots & \vdots \\ 0 & 0 &\ldots & V_{P} \end{array}\right], $$

where *V*_*i*_=*V*(*μ*_*i*_)=*B* ^″^(*θ*_*i*_)=*G* ^′^(*θ*_*i*_), *i*=1,…,*N*, and let $\tilde {Y} = [Y_{1}- B'({\theta }_{1}), \ldots, Y_{N} -B'({\theta }_{N})]^{t} = [Y_{1}- \mu _{1}, \ldots, Y_{N} -\mu _{N}]^{t}$, we have

4 $$\begin{array}{*{20}l} \nabla E &= -D^{-1}(DX^{t}\tilde{Y} - {\lambda} {\beta}),  \\ H({\beta}) &= D^{-1}(DX^{t} V X + {\lambda} I). \end{array} $$

The Newton-Raphson iteration for *β* is

$$\begin{array}{*{20}l} {\beta}^{new} &= {\beta}^{old} - \left\{H\left({\beta}^{old}\right)\right\}^{-1}\nabla E \\ & = {\beta}^{old} + \left(DX^{t} V X + {\lambda} I\right)^{-1}\left(DX^{t}\tilde{Y} - {\lambda} {\beta}^{old}\right) \\ & = \left(DX^{t} V X + {\lambda} I\right)^{-1}\left[DX^{t}VX {\beta}^{old} + DX^{t}\tilde{Y}\right] \\ & = \left(DX^{t} V X + {\lambda} I\right)^{-1}DX^{t}\left[VX {\beta}^{old}+ \tilde{Y}\right]. \end{array} $$

Let $Z = VX {\beta }^{old}+ \tilde {Y}$, we have

5 $$\begin{array}{*{20}l} {\beta}^{new} &= \left(DX^{t} V X + {\lambda} I\right)^{-1}DX^{t} Z,  \\ \eta &= {\beta}^{old} = {\beta}^{new}. \end{array} $$

Different link functions will lead to different regression models as shown as in Table 1.

**Table 1:** Link functions for linear, logistic and Poisson regression models in GLM, where different models have different *A*(∗), *B*(∗), and *C*(∗)

GLM models	*B*(*θ*)	*μ*(*θ*)=*B* ^′^(*θ*)	Link *θ*(*μ*)	*V*(*μ*)=*B* ^″^(*θ*)
Linear regression	*θ* ^2^/2	*θ*	Identity	1
Logistic regression	log(1+*e* ^*θ*^)	$\frac {1}{1 +e^{-{\theta }}}$	logit	*μ*(1−*μ*)
Poisson regression	exp(*θ*)	exp(*θ*)	log	*μ*

Other GLMs such as negative binomial, gamma, and inverse Gaussian can be implemented accordingly with a different *V*(*μ*). When dealing with big data problems with *N*≪*P*,where *N* is the number of samples and *P* is the number of parameters, the inverse of a *P*×*P* matrix is time-consuming and computational challenging. We proposed an efficient algorithm to calculate the inverse of a much smaller *N*×*N* matrix as follows (Liu et al. 2015):

$$ \left(DX^{t} V X + {\lambda} I_{P\times P}\right)^{-1}DX^{t} = DX^{t}\left(VXDX^{t} + {\lambda} I_{N\times N}\right)^{-1}. $$

So that when *N*≪*P*, we have a much efficient estimation:

6 $$\begin{array}{*{20}l} {\beta}^{new} &= DX^{t}\left(V XDX^{t} + {\lambda} I\right)^{-1}Z,  \\ \eta &= {\beta}^{old} = {\beta}^{new}. \end{array} $$

The adaptive ridge algorithm (*L*_0_ADRIDGEA) is implemented in MATLAB are as follows:



The algorithm is easy to implement and very efficient for either small sample size and large dimension or large sample size and small dimension big data problem. The regularized parameter *λ* can be determined either by cross-validation or by AIC and BIC with *λ*=2 and *λ*= log(*N*), respectively. We further discuss that the proposed method is a *L*_0_ approximation and converges to *L*_0_ when the number of iterations *m*→*∞*.

**Algorithm justification:** Given a high-dimensional big feature matrix *X*_*N*×*P*_(*N*≪*P*) and a threshold *γ* for the coefficient estimates, *L*_0_ rejects all the coefficient estimates below *γ* to 0 and keeps the large coefficients unchanged. This is the same as defining a binary vector *s*=[…,1,0,…,1]^*t*^, with the value of 0 or 1 for each feature, where *s*_*j*_=1 if the coefficient estimate for that feature is above the threshold *γ*, and 0 otherwise. Let *S*=diag(*s*) be a matrix with *s* on its diagonal, we have the selected feature matrix *X*_*S*_=*XS*. We can build the standard models with the matrix *X*_*S*_, if we know *s* in advance. For instance, we can estimate the coefficients of a GLM with *L*_2_ regulation given *X*_*S*_ and *Y* with

7 $$ {\beta}^{new} = (X_{S}^{t}VX_{S} + {\lambda} I)^{-1}X_{S}^{t} Z = (X_{S}^{t}VX + {\lambda} I)^{-1}X_{S}^{t}Z =(SX^{t}VX + {\lambda} I)^{-1}SX^{t}Z,  $$

where $Z = VX {\beta }^{old}+ \tilde {Y}$, $\tilde {Y} = [Y_{1}- \mu _{1}, \ldots, Y_{N} -\mu _{N}]^{t}$, and $X_{S}^{t}VX_{S} = SX^{t}VXS = SX^{t}VX$ because of the special structure of matrix *S*. It is guaranteed that the estimate is 0 for feature *j* with *s*_*j*_=0. However, in reality we do not know *s*. Estimating both *s* and *θ* is an NP-hard problem, since we need to solve a mixed-integer optimization problem. Comparing Eq. (7) with Eq. (5), *β* ^*new*^=(*DX* ^*t*^ *VX*+*λI*)^−1^ *DX* ^*t*^ *Z*, it is clear that *S* is replaced by *D* and a binary *s*_*j*_ is approximated by a continuous $\eta _{j}^{2}$ in proposed algorithm. Therefore, the proposed method is a *L*_0_ approximation.

Recall the iterative system in Eq. (3), note that each feature is penalized by a different penalty, which is inversely proportional to the squared magnitude of that parameter estimator *η*_*j*_. i.e.,

$$ {\lambda}_{j} = \frac{ {\lambda} }{2\eta_{j}^{2}}, \;\;\textrm{and }\;\; \eta_{j} = {\beta}_{j}. $$

Smaller *β*_*j*_ will lead to larger *λ*_*j*_. A tiny *β*_*j*_, will become smaller and *λ*_*j*_ will be getting larger in each iteration of *L*_0_ADRIDGE algorithm. *β*_*j*_→0, and *λ*_*j*_→*∞*. On the other hand, a larger *β*_*j*_ will lead a finite *λ*_*j*_, and nonzero *β*_*j*_, when the number of iteration goes to *∞*. The solution of *L*_0_ADRIDGE will converge to that of Eq. (7), because the effect of nonzero *η*_*j*_ will be canceled out in Eq. (5). Note that our proposed methods will find a sparse solution with a large number of iterations and small *ε*, even though the solution of *L*_2_ regularized modeling is not sparse. Small parameters (*β*_*j*_s) become smaller at each iteration and will eventually go to zero (below the machine *ε*). We can also set a parameter to 0 if it is below predefined *ε*=1*e*−6 to speed up the convergence of the algorithm.

## Results

### Simulations

**Poisson Regression:** Our first simulation was used to evaluate the performance of our method for high dimensional Poisson regression. The data was generated from Poisson distribution with different sample sizes (N) and dimensions (P). However, only features 1, 5, 10 and the constant term are used to generate the Poisson counts with [*β*_0_,*β*_1_,*β*_5_,*β*_10_]=[1,0.5,0.5,0.4]. The count *Y* is generated with *Y*=*Poisson*(*μ*), where mean *μ*= exp(*β* *X*). The proposed method is compared with the glmnet ([16] and SparseReg package [17, 18]. glmnet and SparseReg implemented the elastic net, SCAD, and MC+ penalties with an efficient path algorithm. We compare the performance of our approach with *L*_1_ (glmnet), SCAD and MC+ using the popular BIC (*λ*= log(*N*)) criteria. Our *L*_0_ADRIDGE is compared to the glmnet for *L*_1_ and SparseReg for both SCAD and MC+. The results of different methods are presented in Table 2.

**Table 2:** Performance of different GLM methods for Poisson regression over 100 simulations, where values in the parenthesis are the standard deviations, and ANSF: Average number of selected features; rMSE: Average square root of mean squared error; $|\hat { {\beta } }- {\beta } | =\sum _{i} |\hat { {\beta } }_{i} - {\beta }_{i}|$: average absolute bias when comparing true and estimated parameters

	PMS	glmnet	SparseReg		*L*_0_ADRIDGE
		*L*_1_	SCAD	MC+	
	rMSE	1.10(±.091)	1.090(±.092)	**1** **.** **0** **8** **7** **(** **±** **.** **0** **9** **1** **)**	1.937(±.222)
N =100	$|\hat { {\beta }} - {\beta } |$	1.755(±.274)	1.754(±.275)	1.737±.273)	**0** **.** **2** **2** **2** **(** **±** **.** **1** **1** **6** **)**
P =100	ANSF	43.03(±3.52)	43.07(±3.57)	42.06(±3.51)	**3** **.** **9** **9** **(** **±** **.** **1** **0** **0** **)**
	PTM	0*%*	0*%*	0%	**9** **9** **%**
	FDR	90.6*%*	90.6*%*	90.6*%*	**0** **%**
	rMSE	0.503(±.017)	0.502(±.017)	**0** **.** **5** **0** **1** **(** **±** **.** **0** **1** **8** **)**	2.108(±.359)
N =100	$|\hat { {\beta }} - {\beta } |$	2.671(±.421)	2.673(±.425)	2.821±2.012)	**0** **.** **4** **2** **4** **(** **±** **.** **3** **5** **0** **)**
*P*=10^3^	ANSF	75.47(±5.61)	75.82(±5.71)	75.14(±8.69)	**3** **.** **6** **1** **0** **(** **±** **.** **6** **0** **1** **)**
	PTM	0*%*	0*%*	0*%*	**6** **4** **%**
	FDR	94.7*%*	94.7*%*	94.6*%*	**2** **.** **4** **%**
	rMSE	**0** **.** **2** **7** **1** **(** **±** **.** **0** **0** **4** **)**	0.272(±.012)	0.275(±.025)	1.916(±.081)
N =500	$|\hat { {\beta }} - {\beta } |$	5.845(±.280)	6.185(±2.359)	5.807±.273)	**0** **.** **0** **8** **6** **(** **±** **.** **0** **3** **3** **)**
*P*=10^4^	ANSF	465.6(±14.1)	475.1(±15.5)	463.6(±13.9)	**4** **.** **0** **0** **0** **(** **±** **.** **0** **0** **0** **)**
	PTM	0*%*	0*%*	0%	**1** **0** **0** **%**
	FDR	99.1*%*	99.2*%*	99.1*%*	**0** **%**

PMS: Performance Measures. PTM: Percentage of true models. FDR: False discovery rate. The values in boldface indicate the best performance

Table 2 shows that our *L*_0_ADRIDGE consistently achieved the best performance with BIC and different sample sizes and dimensions. With BIC, although MC+ has the lowest square root of mean squared error (rMSE), and fits the data better, *L*_0_ADRIDGE achieves the least absolute bias $|\hat { {\beta }} - {\beta } |$, highest percentage of identified true model (PTM), and lowest false discovery rate (FDR) under different simulation settings. The average number of selected features (ANSF) with *L*_0_ADRIDGE is also closest to the true number 4. Particularly, *L*_0_ADRIDGE found 100% true model with the lowest average absolute bias (0.086) under the dimension of *P*=10,000 and sample size of *N*=500, indicating that the proposed approach is efficient under extra-high dimensional setting. Another interesting finding is that the square root of mean squared errors and absolute biases with *L*_0_ADRIDGE did not vary much across different simulation setting, indicating the robustness of the proposed approach. Moreover, *L*_0_ADRIDGE with BIC is slightly faster than different routines implemented in glmnet and SparseReg in computational time. Finally, BIC apparently is not a good model selection criteria for *L*_1_, SCAD and MC+. More features are selected than necessary. A larger *λ* is needed for selecting the correct model. We reported the results with a larger *λ* on Additional file 1: Table S1, and demonstrated that both SCAD and MC+ can achieve a much smaller FDR, but a larger absolute bias and rMSE.

**Logistic regression:** The logistic regression data was generated with the coefficients of [*β*_1_,*β*_5_,*β*_10_]=[0.5,0.5,−0.4], respectively, and the remaining coefficients were set to zero. The score *z*=*X* *β*+*ε*, where *ε* is the random noise with the signal to noise ratio of 4. Then, the probability *y* is generated from the logistic function *y*=1/(1+*e* ^−*z*^). Note that *y* is the true probability instead of binary (1/0) in this simulation. Unlike the previous example, the optimal values of *λ* in this simulation were selected with the standard 5-fold cross-validation. We divided the *λ* from *λ*_min_=1*e*−4, to *λ*_max_ into 100 equal intervals in log-scale, then chose the optimal *λ* with the smallest test error. The simulation was also repeated 100 times. The computational results were reported in Table 3. The values in the parenthesis are the positive/negative standard deviation.

**Table 3:** Performance of different GLM methods for logistic regression over 100 simulations, where ANSF: Average number of selected features; trMSE: Test Average square root of mean squared error; $|\hat { {\beta } }- {\beta } | =\sum _{i} |\hat { {\beta } }_{i} - {\beta }_{i}|$: average absolute bias when comparing true and estimated parameters

	PMS	*L*_1_	SparseReg		*L*_0_ADRIDGE
			SCAD	MC+	
	trMSE	0.0474(±.0035)	0.0469(±.0039)	0.0456(±.0042)	**0** **.** **0** **4** **3** **4** **(** **±** **.** **0** **0** **2** **8** **)**
N =100	$|\hat { {\beta }} - {\beta } |$	0.2984(±.1262)	0.3129(±.1249)	0.1625(±.0752)	**0** **.** **0** **6** **8** **2** **(** **±** **.** **0** **4** **1** **6** **)**
P =100	ANSF	17.10(±9.32)	18.35(±10.185)	10.410(±6.174)	**3** **.** **3** **3** **0** **(** **±** **.** **7** **7** **9** **)**
	PTM	0%	0*%*	2*%*	**8** **1** **%**
	FDR	77.7*%*	78.4*%*	62.4*%*	**6** **.** **6** **%**
	trMSE	0.0517(±.0045)	0.0496(±.0046)	0.0468(±.0045)	**0** **.** **0** **4** **3** **4** **(** **±** **.** **0** **0** **3** **0** **)**
N =100	$|\hat { {\beta }} - {\beta } |$	0.5968(±.2599)	0.6465(±.2205)	0.2818(±.1030)	**0** **.** **0** **7** **5** **4** **±** **.** **0** **6** **0** **0** **)**
*P*=1000	ANSF	50.92(±39.974)	73.030(±40.792)	24.80(±13.314)	**3** **.** **4** **1** **(** **±** **1** **.** **0** **6** **5** **)**
	PTM	0%	0*%*	0*%*	**8** **0** **%**
	FDR	90.5*%*	93%	83.9*%*	**7** **.** **3** **%**

PMS: Performance Measures. PTM: Percentage of true models. FDR: False discovery rate. The values in boldface indicate the best performance

Table 3 shows that *L*_0_ADRIDGE outperforms *L*_1_, SCAD and MC+ with a substantial margin under the 5-fold cross-validation. Cross-validation is a standard tool for parameter selection in machine learning. *L*_0_ADRIDGE achieved the smallest test square root of mean squared error, least absolute biases, the lowest FDR, and highest percentages of identified true models The average number of selected features are 3.33 and 3.41 for the dimensions of 100 and 1000, respectively, which are the closest to the true number of features 3. In contrary, *L*_1_, SCAD and MC+ selected unnecessary features. *L*_1_ on average identified 17.1 and 50.92 features, and SCAD selected 18.35 and 73.03 features on average for the dimensions of 100 and 1000, respectively, while MC+ performed slightly better, choosing 10.41 and 24.8 features for the dimensions of 100 and 1000, respectively. More impressively, out of 100 simulations, *L*_0_ADRIDGE identified the true model 81 and 80 times with different dimensions, while *L*_1_ and SCAD could not find the true model once, and MC+ only identified the true model 2 times for the dimension of 100, indicating the super performance of *L*_0_ADRIDGE under cross-validation. Finally, *L*_0_ADRIDGE is robust. The test square root of mean squared error and other performance measures did not vary much when the dimension increased from 100 to 1000. It is worth noting that our proposed method performs well with the popular statistical model selection criteria such as BIC and cross-validation. Other popular methods such as *L*_1_, SCAD, and MC+ select more features than necessary with such criteria. Therefore, many popular packages including the commercial MATLAB usually choose a larger *λ* one standard deviation above the minimum test error with cross-validation, which is arbitrary and leads to larger bias. To overcome such bias in parameter estimation, some packages re-estimate the parameters with the selected features and standard GLM model. Unlike these methods, our proposed method performed much better without any postprocessing. Finally, the algorithm is very robust with different initialization. With *N*=100, *P*=1000 and 100 times of different randomized initialization, we achieved the trMSE of 0.437(±.003), average absolute bias of 0.0763(±.07), ANSF of 3.39(±1.154), PTM of 85% and FDR of 6.9*%*, which is quite similar to the results with a fixed initialization.

### TCGA ovarian cancer data

**Fig. 1:**
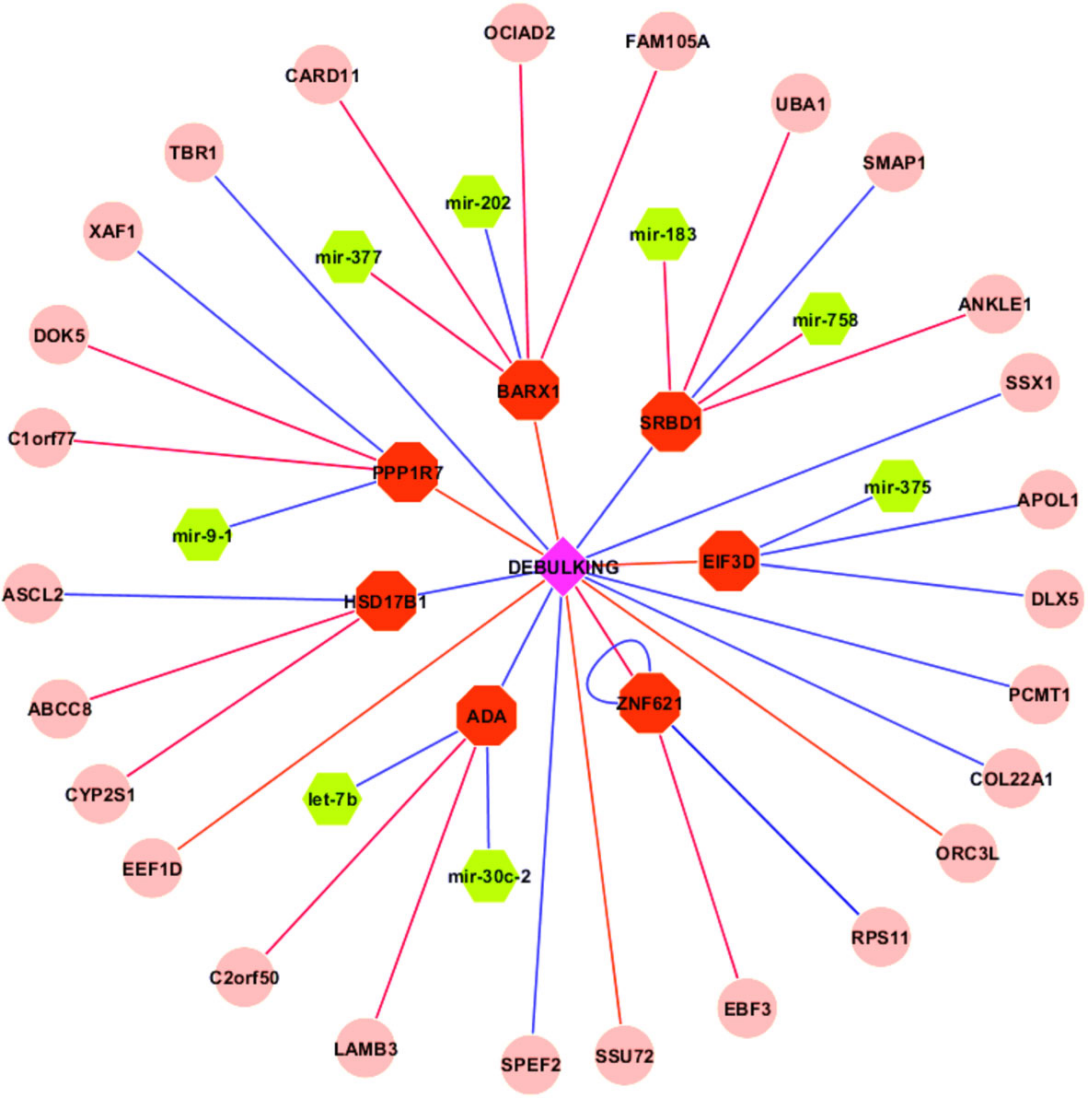
Gene signatures associated with suboptimal debulking, where nodes in red: mRNA signatures; nodes in green: microRNA signatures; nodes in pink: methylation signatures, and edges in red: positive partial correlation; edges in blue: negative partial correlation

The Cancer Genomic Atlas (TCCA) has generated a large amount of next generation sequencing and other omics data for ovarian adenocarcinoma (OC). In this study, we conducted integrated analysis of RNA-seq, miRNA expression, promoter methylation, and debulking status data from 367 OC patients. There are 342 microRNAs, 13,911 mRNA expression (in FPKM), and 21,985 promoter methylation values available. We first normalized different omics data and screened the debulking associated microRNA, mRNAs, and methylation promoters with the *P*-values of less than 0.01 with the training data only. Based on the central dogma of biology, suboptimal debulking is associated with microRNA expression, gene expression, and DNA methylation; gene expression is a function of microRNA expression and DNA methylation; and microRNA expression is regulated by DNA methylation. *L*_0_ Logistic regression was used for suboptimal debulking prediction, while *L*_0_ penalized Poisson regression was used for gene expression and microRNA expression prediction with FPKM. FPKM, representing fragments per kilobase of exon per million fragments mapped, measures the normalized read counts for RNA-seq. Three-fold cross validation was used for gene selection and validation. We reported the gene signatures with the best predicted area under the ROC curves (AUCs). Molecular signatures that are directly or indirectly associated with suboptimal debulking are shown in Fig. 1.

**Fig. 2:**
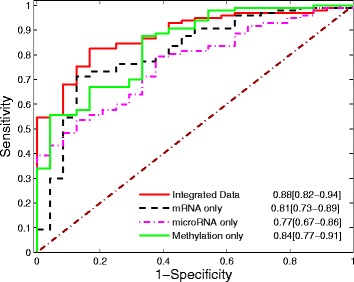
Predictive AUCs for integrated data, mRNA expression only, microRNA expression only, and methylation only

Figure 1 indicates that there are 16 gene signatures including 7 mRNAs and 9 epigenetic markers directly associated with debulking status. Even though there is no microRNA directly associated with debulking, eight microRNA signatures are indirectly associated with debulking through their association with mRNA signatures. Moreover, there are additional 18 epigenetic markers indirectly associated with debulking. The 7 mRNAs directly associated with debulking are EIF3D, PPP1R7, ADA, HSD17B1, SRBD1, ZNF621, and BARX1, where EIF3D, PPP1R7, BARX1 and ZNF621 have positive correlations and the other 3 genes have negative correlations with suboptimal debulking. Among the 7 mRNAs, ADA (Adenosine Deaminase) is a well-studied gene in ovarian neoplasms. ADA levels were found to be significantly higher in patients with ovarian cancers as compared with benign ovarian tumors [19]. ADA has been regarded as a potential biomarker for diagnosis and an agent for the treatment of ovarian cancer [20]. Other mRNAs such as BARX1, EIF3D, PPP1R7, and HSD17B1 are also known to be associated with different cancers or other diseases. At the microRNA level, there are 8 microRNAs indirectly associated with debulking including mir-183, let-7b, mir-9-1, mir-377, mir-202, mir-758, mir-375, and mir-30c-2. While let-7b, mir-30c-2, and mir-377 are positively correlated with suboptimal debulking through mRNAs ADA and BARX1 indirectly, the other 5 microRNAs have indirectly negative correlations with suboptimal debulking. Seven of eight microRNAs except for mir-758 are known to be associated with ovarian cancer. Particularly, let-7b is known to be an unfavorable prognostic biomarker and predict of molecular and clinical subclasses in high-grade serous ovarian carcinoma, and it may also be useful for discriminating between controls and patients with serous ovarian cancer [21, 22]. Mir-183 is known to be associated with multiple cancers. It regulates target oncogene (Tiam1), and reduce the migration, invasion and viability of ovarian cancer cells [23]. Finally, at the DNA level, nine epigenetically modified genes directly associated with debulking are SSX1, TBR1, ZNF621, ORC3L, COL22A1, SPEF2, SSU72, EEF1D, and ZNF621, where EEF1D, SSU72, and ORC3L are positively associated with suboptimal debulking, while 6 other epigenetic genes are negatively correlated with suboptimal debulking. In addition, 18 other epigenetic genes indirectly associated with debulking may also have biological implications. Finally, integration of multi-omic data increases the prediction power substantially. Besides analyzing three types of omics data together, we performed the same three-fold cross validation for gene expression, methylation, and microRNA expression separately. The AUC curves are in Fig. 2.

Figure 2 shows that the best predicted AUC over 100 simulations for integrated data is 0.88, while the best predictive AUCs for gene expression, methylation, and microRNA over 100 simulations are 0.81, 0.84, and 0.76, respectively. The AUC with integrated data achieved the highest AUC, indicating the importance of multi-omics data mining. Genes selected with mRNA, microRNA, and methylations separately are reported in the supplementary document. In addition, we also compare the selected features and the same number of top genes identified with statistical test. The results are reported on Additional file 1: Table S2, and demonstrate that although individual genes are more statistically significant, combination of a panel of genes with standard logistic regression has less predictive power and test AUC (0.79).

## Conclusions

Biomarkers from multi-omics data may predict disease status and help physicians to make clinical decisions. *L*_0_ based GLM, which directly penalizes the number of nonzero parameters, has nice theoretical properties and leads to essential sparsity for biomarker discovery. Optimizing the *L*_0_ regularization is a crucial, but difficult problem. We have developed an adaptive ridge algorithm (*L*_0_ADRIDGE) for approximating *L*_0_ penalized GLM. The algorithm is easy to implement and efficient for problems with either an ultra-high dimension and small sample size, or a low-dimension and large sample size. It outperforms the other cutting edge regularization methods including *L*_1_, SCAD and MC+ through simulations. When applied to the integration of multilevel omics data from TCGA and the prediction of suboptimal debulking from ovarian cancer, it can identify a panel of gene signatures achieving the best prediction power. We also demonstrate that prediction power of a model with multi-omics data increases substantially, when comparing with a model with one omics data, indicating the importance of big data mining.

## Additional file

Additional file 1 Table S1. Performance of different GLM methods for Poisson regression over 100 simulations, where values in the parenthesis are the standard deviations, and ANSF: Average number of selected features; rMSE: Average square root of mean squared error; $|\hat {\beta } - \beta | = {\sum \nolimits }_{i}|\hat {\beta } - \beta _{i}|$: average absolute bias when comparing true and estimated parameters. PMS: Performance Measures. PTM: Percentage of true models. FDR: False discovery rates. The L0ADRIDGE is compared to the best performance chosen from *λ*=0.9*λ*_max_ and *λ*=0.5*λ*_*max*_ with both for SCAD and MC+. Table S2. The comparison of performance of the our sparse modeling approach and the top genes selected with Student’s t-test. The results demonstrate that although each gene is more statistically significant with statistical test, the combination of the panel of genes has less predictive power and test AUC with standard logistic regression and three-fold cross valida- tion, indicating the collinearity among theses genes. (PDF 86 kb)

## Abbreviations

AUC: The area under an ROC curve
GLM: Generalized linear model
*L*_0_ADRIDGE: *L*_0_ adaptive ridge algorithms
MC+: Minimax concave penalty. SCAD: Smoothly clipped absolute deviation
TCGA: The cancer genome atlas
