# Supplementary material for: Sparse generalized linear model with L0 approximation for feature selection and prediction with big omics data
Source: BioData Min. 2017 Dec 19;10:39. doi: 10.1186/s13040-017-0159-z (PMC5735537; doi:10.1186/s13040-017-0159-z)
Supplement: Additional file 1 — Table S1. Performance of different GLM methods for Poisson regression over 100 simulations, where values in the parenthesis are the standard deviations, and ANSF: Average number of selected features; rMSE: Average square root of mean squared error; $|\hat {\beta } - \beta | = {\sum \nolimits }_{i}|\hat {\beta } - \beta _{i}|$|β^−β|=∑i|β^−βi|: average absolute bias when comparing true and estimated parameters. PMS: Performance Measures. PTM: Percentage of true models. FDR: False discovery rates. The L0ADRIDGE is compared to the best performance chosen from λ=0.9λ max and λ=0.5λ max with both for SCAD and MC+. Table S2. The comparison of performance of the our sparse modeling approach and the top genes selected with Student’s t-test. The results demonstrate that although each gene is more statistically significant with statistical test, the combination of the panel of genes has less predictive power and test AUC with standard logistic regression and three-fold cross valida- tion, indicating the collinearity among theses genes. (PDF 86 kb) [file 13040_2017_159_MOESM1_ESM.pdf]

Supplementary Table 1:

Performance of different GLM methods for Poisson regression over 100 simulations, where values in the parenthesis are the standard deviations, and ANSF: Average number of selected features; rMSE: Average square root of mean squared error; $|\hat{\beta}-\beta| = \sum_i |\hat{\beta}_i - \beta_i|$: average absolute bias when comparing true and estimated parameters. PMS: Performance Measures. PTM: Percentage of true models. FDR: False discovery rates. The L_0ADRIDGE is compared to the best performance chosen from $\lambda = 0.9\lambda_{\max}$ and $\lambda = 0.5\lambda_{max}$ with both for SCAD and MC+.

	PMS	SparseReg		L_0ADRIDGE	
		SCAD	MC+	AIC	BIC
	rMSE	3.650(±.468)	3.632(±.474)	**1.831**(±**.188**)	1.902(±.227)
N =100	$\lvert\hat{\beta}-\beta\rvert$	1.198(±.203)	1.192(±.204)	0.255 ± .164)	**0.241**(±**.150**)
P =100	ANSF	3.28(±.767)	3.28(±.767)	4.29(±.574)	**3.97**(±**.171**)
	PTM	43%	43%	73%	**97**%
	FDR	**0.2**%	**0.2**%	6.3%	0.25%
	rMSE	3.716(±.471)	3.711(±.470)	**1.882**(±**.198**)	2.164(±.374)
N =100	$\lvert\hat{\beta}-\beta\rvert$	1.157(±.194)	1.153(±.204)	**0.434** ± **.372**)	0.503(±.343)
$P = 10^3$	ANSF	3.49(±.870)	3.49(±.870)	**4.38**(±**.763**)	3.5(±.659)
	PTM	39%	39%	**62**%	58%
	FDR	4.44%	4.44%	12.9%	**2.92**%
	rMSE	3.826(±.268)	3.825(±.268)	**1.890**(±**.0985**)	1.938(±.115)
N =500	$\lvert\hat{\beta}-\beta\rvert$	1.107(±.146)	1.106(±.146)	0.146 ± .067)	**0.086**(±**.035**)
$P = 10^4$	ANSF	3.68(±.471)	3.68(±.471)	5.12(±.872)	**4.000**(±**.000**)
	PTM	68%	68%	24%	**100**%
	FDR	**0**%	**0**%	19.7%	**0**%

Supplementary Table 2

The comparison of performance of the our sparse modeling approach and the top genes selected with Student's t-test. The results demonstrate that although each gene is more statistically significant with statistical test, the combination of the panel of genes has less predictive power and test AUC with standard logistic regression and three-fold cross validation, indicating the collinearity among theses genes.

Data Sources	Sparse Modeling		Top genes (t-test)	
	Gene IDs	coefficients	Gene IDs	P-values
RNA	EIF3D	0.5234	MAST1	1.46E-06
RNA	PPP1R7	0.5315	GTPBP3	3.48E-06
RNA	ADA	-0.4230	TRIM45	3.56E-06
RNA	HSD17B1	-0.5073	L3MBTL	3.83E-06
RNA	SRBD1	-0.5210	SFRP2	7.21E-06
RNA	ZNF621	-0.6236	COL11A1	1.69E-05
RNA	BARX1	0.3515	C19orf57	2.73E-05
Methylation	PCMT1	-0.3630	GAB3	1.45E-06
Methylation	EEF1D	0.6594	HTR6	4.65E-06
Methylation	SSU72	1.0168	DNALI1	7.59E-06
Methylation	SPEF2	-3.8169	SYK	1.57E-05
Methylation	COL22A1	-0.7095	FLRT2	5.80E-05
Methylation	ORC3L	0.4155	PCDH11X	9.09E-05
Methylation	ZNF621	0.1981	KIAA1279	0.00010
Methylation	TBR1	-0.2790	COL22A1	0.00010
Methylation	SSX1	-0.4062	TRPC6	0.00012
	Test AUC =0.88		Test AUC = 0.79	
